# Goal-directed therapy and acute kidney injury: as good as it gets?

**DOI:** 10.1186/s13054-016-1346-x

**Published:** 2016-06-25

**Authors:** James F. Doyle, Marlies Ostermann, Lui G. Forni

**Affiliations:** Intensive Care Unit and Surrey Perioperative Anaesthesia & Critical Care Collaborative Research Group (SPACeR), Royal Surrey County Hospital NHS Foundation Trust, Egerton Road, Guildford, UK; Department of Critical Care, King’s College London, Guy’s and St. Thomas’ Foundation Hospital, London, UK; School of Health Sciences, Faculty of Health & Medical Sciences, University of Surrey, Guildford, UK

**Keywords:** Goal-directed therapy, Enhanced recovery programme, Acute kidney injury

## Abstract

The use of goal-directed therapy as part of an enhanced recovery programme is well established in terms of management of the modern high-risk surgical patient in order to reduce both morbidity and mortality. The mechanisms behind this improvement are debated, but a reduction in the development of post-operative complications including acute kidney injury may be relevant. A recent study examining this relationship has been reported and is discussed here.

Since the initial observation by Shoemaker that post-operative mortality in high-risk surgical patients could be reduced through optimising oxygen delivery, hence limiting the ‘oxygen debt’, much research has focussed on this approach [1–4]. This has evolved to the concept of goal-directed fluid therapy with optimisation of flow-related haemodynamic variables rather than aiming for a fixed value. Such an approach, together with initiatives such as enhanced recovery, has been shown to improve outcomes in patients undergoing major abdominal surgery [5, 6]. However, major non-cardiac surgery still remains associated with significant post-operative complications which correlate with survival. Of these, the development of acute kidney injury (AKI) is independently associated with both short-term and long-term complications [7, 8]. A recent systematic review examined the incidence and associations of AKI after major abdominal surgery and demonstrated that the pooled incidence of AKI was 13.4 %, with length of stay and non-renal post-operative complications increased in those developing AKI [9]. In studies reporting short-term mortality, the relative risk of death in the presence of post-operative AKI was 12.6-fold.

It follows that the development of strategies to reduce post-operative AKI may translate into improved outcomes, which provides the basis of the study performed by Schmid and co-workers in this issue of the journal [1]. This single-centre, randomized controlled study examined whether the use of an algorithm-guided goal-directed haemodynamic therapy (GDT) using transpulmonary thermodilution could improve renal outcomes compared to standard practice within their institution. End-points included post-operative complications, 1-year mortality and, as the primary end-point, change in measured serum creatinine. One hundred and ninety three patients were randomized, with 92 assigned to the GDT group and 88 to the control group; 13 were excluded from analysis. Transpulmonary thermodilution measurements were carried out every 30 min during anaesthesia with global end-diastolic index (GEDI), mean arterial pressure (MAP), and cardiac index (CI) used to assess whether the algorithm objectives were met. Where a fluid bolus was required, 500 ml hydroxyethyl starch (HES; 130/0.4 to a maximum dose of 50 ml/kg bodyweight per day) or balanced crystalloid were used over a 15-min period. Norepinephrine was used as a vasopressor, and dobutamine as an inotrope. In the control group, only invasive blood pressure monitoring was applied but a second anaesthetist performed transpulmonary thermodilution after induction and then every 30 min as in the intervention arm. The attending physician was blinded to the data. Similarly, the results were blinded during the post-operative period in the intensive care unit (ICU).

So, what did the results show? Firstly there was no significant difference in post-operative delta creatinine. Also, there was no difference in post-operative complications, hospital mortality, or 1-year mortality between the groups. At first glance one may assume this is yet another nail in the goal-directed therapy coffin, but perhaps this is not the case. A novel aspect of this study is the fact that haemodynamic parameters were measured in both groups and, interestingly, achievement of haemodynamic goals was not lower in the control group. In fact >80 % of the control group met the target values. Therefore, the algorithm added little above and beyond standard care, in keeping with some of the recent studies on the use of goal-directed therapy in septic shock [10], or following upper gastro-intestinal surgery such as in the OPTIMISE study, where additionally there was minimal difference between total fluid volume infusion between groups [4]. Subsequent multivariate regression analysis of the work by Schmid et al. identified intra-operative hypotension (MAP <70 mmHg) and post-operative hypovolaemia (GEDI <640 ml/m^2^) as risk factors for post-operative renal impairment, as was the use of HES. Whilst there was no difference between groups in the volume infused or noradrenalin rates, the peri-operative use of dobutamine was significantly higher in the intervention group. There is evidence that dobutamine-induced vasodilatation can worsen renal perfusion [11], and one may therefore question the choice of inotrope used.

Unsurprisingly there was no effect on renal outcomes with the use of the algorithm, although renal impairment was found in over 50 % of both groups when defining AKI by the Risk Injury Failure Loss Endstage (RIFLE) or Kidney Disease Improving Global Outcomes (KDIGO) criteria. If serum creatinine alone were used for AKI determination, the incidence fell to 27 % and 24 %, respectively. This in itself raises the issue as to how post-operative AKI be defined. A recent retrospective analysis of electronic health records from over 30,000 patients confirmed that not only does mortality increase with increasing AKI severity but that when both serum creatinine and urine output criteria are fulfilled in-hospital mortality rises above 50 % [12]. Interestingly, surgical admissions were far more likely to have AKI diagnosed on urine output alone, with duration of AKI shorter when only urinary criteria were applied which may reflect physiological oliguria rather than functional damage in this select group.

Does this mean that goal-directed therapy offers no advantage in terms of reducing AKI? Obviously no such conclusion can be drawn from this study given the lack of differences between the two groups. There is clear evidence that haemodynamic management remains important to prevent AKI. The study by Schmid et al. and other studies demonstrated that intra-operative hypotension (MAP <55 mmHg) is an independent risk factor for subsequent AKI [1, 13]. Moreover, studies in critically ill patients with early AKI demonstrated that a higher oxygen delivery and MAP were independently associated with a lower risk of progression in terms of AKI, implying a pivotal role of oxygen delivery and utilisation [14]. Clearly the use of injudicious volume therapy in whatever clinical arena is unwise as supported by a considerable body of evidence [15].

The trial by Schmid et al. demonstrates that, even in the absence of specific haemodynamic optimisation, overall care of the high-risk surgical patient seems to have improved. Perhaps the future role of GDT is in the prevention of volume depletion or overload. This might be facilitated by the development of a volume therapeutic index, a proposed next target in the field of critical care nephrology.

## Abbreviations

AKI, acute kidney injury; CI, cardiac index; GEDI, global end-diastolic index; GDT, goal-directed haemodynamic therapy; HES, hydroxyethyl starch; MAP, mean arterial pressure
